# PP30 The Health System Impact Of Returning Rare Disease Variants As Secondary Findings From Genomic Sequencing: A Population-Based Model

**DOI:** 10.1017/S0266462324002034

**Published:** 2025-01-07

**Authors:** Nick Dragojlovic, Nicola Kopac, Elisabet Rodriguez Llorian, Anna Lehman, Larry D. Lynd

## Abstract

**Introduction:**

The American College of Medical Genetics and Genomics (ACMG) recommends that pathogenic variants linked to 37 genetic diseases be disclosed as secondary findings (SFs) to patients undergoing genome-wide sequencing (GWS), including rare diseases (RDs) treated with enzyme replacement or gene silencing/replacement therapies. We estimated the potential budget impact of treating these RD patients in the US and Canada.

**Methods:**

A population-based model was used to estimate the number of patients that would be identified in a given jurisdiction for the 11 diseases on the ACMG’s SFv3.2 list classified as “inborn errors of metabolism” or “miscellaneous phenotypes.” Genetic and clinically diagnosed prevalence was estimated for each gene-phenotype pair based on the scientific literature. Demographic and GWS utilization data were obtained from government websites and payer reports. Drug costs were obtained from the literature and payer websites and reported in 2023 US dollars. A range of one-way sensitivity and scenario analyses were conducted, and province- and state-level estimates were also generated.

**Results:**

Based on an estimated current annual GWS utilization rate of 77 per million in the US, 237 RD SFs would be identified annually (0.9% of 25,663 patients tested), out of which 107 (45%) would be variants linked to hereditary transthyretin amyloidosis (hATTR). In Canada (GWS utilization rate of 169 per million), 45 patients would be identified per year, of which 11 (24%) would be hATTR. Treating 50 percent of hATTR SF patients with patisiran would cost approximately USD26.0 million in the USA and USD2.8 million in Canada annually. In contrast, no cases of RPE65-related retinopathy would be detected at current utilization rates.

**Conclusions:**

The addition of hATTR (with an estimated genetic prevalence of one in 240 in the US) to the ACMG SF list may result in a significant number of additional cases diagnosed per year. While there is an ongoing debate about initiating presymptomatic treatment, reimbursement requests for high-cost drugs like patisiran are likely to increase if GWS utilization continues to grow.

